# Modulation factors calculated with an EPID‐derived MLC fluence model to streamline IMRT/VMAT second checks

**DOI:** 10.1120/jacmp.v14i6.4274

**Published:** 2013-11-08

**Authors:** Stephen Steciw, Satyapal Rathee, Brad Warkentin

**Affiliations:** ^1^ Department of Medical Physics Cross Cancer Institute Edmonton AB Canada; ^2^ Department of Oncology Medical Physics Division University of Alberta Edmonton AB Canada

**Keywords:** modulation factor, IMRT, VMAT, EPID, tongue and groove, verification

## Abstract

This work outlines the development of a robust method of calculating modulation factors used for the independent verification of MUs for IMRT and VMAT treatments, to replace onerous ion chamber measurements. Two‐dimensional fluence maps were calculated for dynamic MLC fields that include MLC interleaf leakage, transmission, and tongue‐and‐groove effects, as characterized from EPID‐acquired images. Monte Carlo‐generated dose kernels were then used to calculate doses for a modulated field and that field with the modulation removed at a depth specific to the calculation point in the patient using in‐house written software, Mod_Calc. The ratio of these two doses was taken to calculate modulation factors. Comparison between Mod_Calc calculation and ion chamber measurement of modulation factors for 121 IMRT fields yielded excellent agreement, where the mean difference between the two was −0.3%±1.2%. This validated use of Mod_Calc clinically. Analysis of 5,271 dynamic fields from clinical use of Mod_Calc gave a mean difference of 0.3%±1.0% between Mod_Calc and Eclipse‐generated factors. In addition, 99.3% and 96.5% fields pass 5% and 2% criteria, respectively, for agreement between these two predictions. The development and use of Mod_Calc at our clinic has considerably streamlined our QA process for IMRT and RapidArc fields, compared to our previous method based on ion chamber measurements. As a result, it has made it feasible to maintain our established and trusted current in‐house method of MU verification, without resorting to commercial software alternatives.

PACS numbers: 87.55.km, 87.55.Qr, 87.55.kd, 87.57.uq

## I. INTRODUCTION

Patient‐specific verification of dose distributions calculated by a clinical treatment planning system (TPS) remains an important responsibility of the medical physicist.^(1)^ This verification may incorporate computational or measurement techniques, or both. As outlined in the Appendix of the report of AAPM Task Group 114,^(1)^ a common approach for conventional conformal radiotherapy fields is to validate the monitor units (MUs) for a given field based on an independent calculation of the dose in a simple phantom geometry using standard dosimetric factors (e.g., collimator scatter factor, phantom scatter factor, and tissue phantom ratio) and relevant correction factors accounting for specifics of the patient geometry (e.g., inhomogeneities). For complex intensity‐modulated radiotherapy (IMRT) treatments, independent dose validation typically involves more intensive methods. Many clinics rely on validation using measurements of planar dose distributions using film, diode or ion chamber arrays, or electronic portal imaging devices (EPIDs).^(2)^ Methods of independent computation of IMRT fluence or dose distributions have also been developed.^3^, ^4^, ^5^, ^6^, ^7^, ^8^, ^9^, ^10^, ^11^, ^12^, ^13^, ^14^, ^15^ These employ a variety of approaches that cover a significant spectrum of complexity. Modified Clarkson integrations have been used to estimate the scatter contributions for IMRT fields using mathematical expressions solely based on standard dosimetric factor data.^(^ ^3^ ^,^ ^6^ ^)^ An example of this is the algorithm of Kung et al.,^(3)^ which has been incorporated in the commercial IMRT second check software RadCalc (Lifeline Software, Austin TX) and IMRT Check (Oncology Data Systems, Oklahoma City, OK). Other investigators have integrated a factor‐based calculation with explicit modeling of the scatter contributions ‐ e.g. the exponential model of Baker et al.^(10)^, and the three‐source model developed by Yang et al.^(^ ^7^ ^,^ ^16^ ^)^ and used in the commercial IMSure QA (Standard Imaging, Middleton, WI) software. Pencil beam convolution algorithms^(^ ^5^ ^,^ ^15^ ^,^ ^17^ ^,^ ^18^ ^)^ and Monte Carlo simulations^(^ ^9^ ^,^ ^11^ ^,^ ^13^ ^,^ ^14^ ^)^ have also been implemented to provide a second check of IMRT dose calculations by a TPS.

Although the use of independent IMRT computations may obviate the need for patient‐specific IMRT QA measurements, only such measurements validate the IMRT plan deliverability prior to the patient treatment.^(2)^ For this reason, our institute employs both pretreatment EPID portal dosimetry using Varian software (Varian Medical Systems, Palo Alto, CA) and an independent check of the MUs of IMRT plans. This report is focused on improvements to the latter component. When IMRT was implemented at our clinic, we extended the straightforward factor‐based MU calculation that is performed for our conformal treatments using in‐house software. This required determination of the modulation factor, which is the ratio of doses in the dynamic and corresponding open fields. This factor is estimated based on dose calculations made within our TPS. For TPS‐independent validation of its value, ion chamber measurements of the modulation were made in a solid water phantom. However, these measurements became increasingly onerous as our IMRT workload increased. Thus, the main motivation of this work was to streamline our second‐check process by replacing the ion chamber measurements with an accurate and independent calculation of the modulation factors.

As is reasonable for the requirements of a second‐check software, we pursued a modulation calculation algorithm that provides sufficient accuracy without excessive model complexity and commissioning requirements. The method described in this work is a pencil beam convolution using a spatially invariant, tilt‐free kernel to calculate dose from independently calculated fluence. Our approach uses a water kernel solely derived from Monte Carlo simulations. Thus, the kernel does not require “commissioning,” distinguishing it from previous pencil beam approaches (for independent IMRT calculation).^(^ ^5^ ^,^ ^12^ ^,^ ^15^ ^,^ ^19^ ^)^ Our method also implements a comprehensive modeling of the 2D fluence output of the MLC sequence for each IMRT field.^(20)^ In addition to primary fluence, our fluence modeling incorporates a head scatter model accounting for extrafocal radiation and collimator backscatter, as well as MLC transmission, interleaf leakage, and tongue‐and‐groove effects. The incorporation of MLC interleaf leakage and tongue‐and‐groove effects is a particularly attractive feature since clinical treatment planning systems may approximate these effects to expedite dose calculations. Since these simplifications in the TPS calculations are routinely evident in our portal dosimetry IMRT QA, it is advantageous that our independent check of the modulation factor also be able to identify this as a source of discrepancy. Similar to the approach described by Rosca and Zygmanski,^(21)^ our algorithm uses EPID measurements to accurately characterize the spatial distribution of interleaf and tongue‐and‐groove effects. An advantage of this technique is that commissioning of the fluence modeling to include these effects is relatively straightforward and requires only a small number of EPID measurements; it also does not require detailed knowledge of the geometry of the MLC leaves, as would be necessary if the modeling were based on analytic equations or Monte Carlo simulations. To our knowledge, the use of such an EPID‐based approach specifically for developing independent IMRT second‐check software has not been described in the literature.

The purpose of this report is twofold. The first is to describe details of our modulation factor calculation algorithm, which has been implemented in a new in‐house software called Mod_Calc. As suggested above, the algorithm represents a unique amalgamation of existing approaches in the literature to independent IMRT dose calculation and modeling of the various fluence components. While tailored to the specific needs of our clinic, our approach is of potential interest to other centers, particularly those wishing to adapt existing second‐check calculations of conformal treatments that are based on “hand‐calculations” (i.e., factor‐based) for IMRT treatments. The second purpose is to investigate the performance and versatility of the algorithm to validate its suitability for clinical implementation. This is based on an analysis of nearly 800 sliding window IMRT plans and preliminary results from our first clinical RapidArc plans (Varian Medical Systems, Palo Alto, CA).

## II. MATERIALS AND METHODS

At our center, radiation delivery and treatment planning are performed with Varian equipment (Varian Medical Systems). A 2100EX linear accelerator at 6 MV at a dose rate of 400 MU/min, using a Millenium‐120 multileaf collimator (MLC) was used in this study. Our treatment planning system is Eclipse AAA V10.0.

The EPID images acquired in this work used a Varian aS500 EPID using an active matrix containing 512×384 elements (pixel size=784μm) and an IAS3 acquisition system. EPID images were acquired with 14‐bit precision using Varian's Varis Portal‐Vision (version 6.1) software using integration mode, at a source‐to‐detector distance (SDD) of 105 cm, with no external buildup added to the detector. Flood and dark fields were acquired prior to each image acquisition, and all EPID images have been flood‐ and dark field‐corrected automatically by the Portal‐Vision software.

Ion chamber measurements were conducted at a depth d in solid water with 15 cm of back‐scatter using a Protea ion chamber (model TDC‐100, Protea Systems Corporation, Benicia, CA). This cylindrical ion chamber has an active volume measuring 4 mm in diameter and 11 mm in length.

Monte Carlo pencil beam dose kernels were simulated with 100 million histories using EGSnrc/DOSXYZnrc software. The PRESTA‐II algorithm was used with cutoff values of ECUT=0.521MeV,andPCUT=0.010MeV. The kernels were scored in a $30\times 30\;{\text{cm}}^{2}$ water phantom at the depth of interest with 15 cm of backscatter, using a 6 MV incident linac spectrum.^(22)^ The scoring plane was sampled at 784μm in both directions.

The MATLAB programming language (The MathWorks Inc., Natick, MA) was used throughout this work. This includes the postprocessing of images, the optimization of various parameters, the programming and compiling of the Mod_Calc and Phys_calc programs, and the data analysis that followed.

### A. Mucalc: existing in‐house software to validate MUs

The existing in‐house MUcalc software used to perform the independent check of the MUs of each field is written using the Microsoft Excel VBA environment. The program essentially implements an automated “hand calculation” based on multiplication of standard dosimetric factors (e.g., tissue phantom ratios, head scatter factors). For conformal fields, MUcalc calculates the dose per MU to a chosen reference point ($D_{\text{RefPt}}$) using:

(1) $$D_{re\text{fP}t}=D_{cal}\cdot ISL\cdot S_{c}\cdot S_{p}\cdot TPR\cdot OAR\cdot ICF\cdot WF$$

In the above formula: $D_{\text{cal}}$ is the calibration dose (1 cGy/MU for $10\times 10\;{\text{cm}}^{2}$ field at $d_{\text{max}}$); $S_{c}$ and $S_{p}$ are the collimator and phantom scatter factors, respectively; TPR is the tissue phantom ratio; ISL is the inverse‐square‐law correction; OAR is the depth‐dependent off‐axis ratio; ICF is an inhomogeneity correction factor estimated based on a ratio of the TMRs evaluated at the effective radiological and physical depths; and WF is the wedge factor (if applicable). In performing this calculation, MUcalc reads in the relevant field parameters (e.g., field size, depth) from a plan report generated by our treatment planning system (TPS), and then interpolates the values of the necessary dosimetric factors from tabulated clinical commissioning data. To calculate the dose to the reference point for dynamic MLC deliveries, Eq. (1) is multiplied by an additional factor, accounting for the modulation. This modulation factor, Mod, is defined as:

(2) $$Mod=\frac{\text{dose}\;\text{at}\;\text{reference}\;\text{point}\;\text{with}\;\text{dynamic}\;\text{MLC}}{\text{dose}\;\text{at}\;\text{reference}\;\text{point}\;\text{in}\;\text{open}\;\text{field}\;(i.e\;\text{no}\;\text{MLC})}$$

The modulation factor is a significant complication to the simple MU verification calculation of Eq. (1), since Mod cannot be looked up in a table or calculated from a very simple analytic expression. In our clinic's process, the value of Mod is estimated from dose calculations made within our TPS. Specifically, prior to running MUcalc, the dosimetrist creates a second temporary plan with the MLCs removed from each field. Mod is then the ratio of the MU/Gy values at the reference point for the two plans (with and without the dynamic MLC), as reported by our TPS. This modulation factor is calculated on the patient geometry. Ion chamber measurements of the modulation in a solid water phantom had provided the independent validation of this value. Agreement within ±5% between measured and TPS‐derived Mod values was deemed clinically acceptable. Unfortunately, these ion chamber measurements typically required between 25 and 45 min per plan, and became an increasingly onerous task. In addition, this second set of measurements was not providing additional benefit over the portal dosimetry analysis in detecting potential problems in the IMRT plans. This motivated our effort to find an alternative model‐based method of validating the point modulation factors. (Note, that using the already acquired portal dose images for this purpose is complicated by the need for significant image postprocessing to account for EPID armscatter and off‐axis effects, and to determine the dose at the patient‐specific depth. Workflow considerations regarding the timing of EPID acquisition and the second check also make this an undesirable approach at our clinic.)

### B. EPID‐response derived from primary and head scatter fluence

The EPID was chosen to characterize the MLC's interleaf leakage and tongue‐and‐groove effects due to its high spatial resolution (784μmpixels) and its large size (30cm×40cm). It was used to establish a comprehensive fluence model and, thus, is key to calculating accurate modulation factors. The stages required to characterize interleaf leakage and tongue‐and‐groove effects and produce an accurate full fluence model for a given IMRT field are outlined in the Results section B to D below. In section E, this fluence is convolved with a kernel to produce the dose in water for both the IMRT field and its open field counterpart. These doses are used to calculate the modulation factor.

Software written in MATLAB was used to simulate linac fluences for dynamic MLC deliveries. By linearly interpolating between leaf position control points found in the MLC control file, the in‐house software determines the field's dynamic leaf positions at 600 time intervals. To emulate the effects of the MLC's rounded leaf design, an additional 1.9 mm dosimetric leaf gap was added to the original gap defined in the MLC file. This is similar to the modeling approach of our TPS, and the value of 1.9 mm is based on TPS‐commissioning measurements. Based on the relationships between the nominal leaf position in the MLC file, the geometric leaf edge, and the dosimetric leaf gap described by Vial et al.,^(23)^ an explicit correction for the leaf position offset (using Varian's mlctable.txt file) is unnecessary using such a rounded leaf model. The ideal MLC 2D fluence $\Phi_{\text{ideal}}$ in units of MUs was simulated dynamically for each field assuming a constant dose rate, where the fluence was integrated in 2D over the delivery. Here, the MUs are proportional to the fraction of time that a point on the 2D map is exposed to fluence (i.e., not under an MLC leaf). For an open 1 MU field, $\Phi_{\text{ideal}}$ would have values of 0 MU under the MLC leaves, and 1 MU elsewhere.

A 2D headscatter model was applied to $\Phi_{\text{ideal}}$ based on the method outlined by Jiang et al.^(24)^ Both headscatter and the backscatter into the monitor ion chambers from the secondary collimators are taken into account. In the Jiang study, the headscatter model calculation uses the physical location of the target, flattening filter, and X and Y jaws in a linac. The bottom of the flattening filter is assumed to be the source of the extrafocal radiation. In the Jiang model, the relative intensity of extrafocal fluence is defined by a sum of three Gaussian functions. The Gaussian parameters are optimized based on measured central axis headscatter factors. In the present work, square field sizes of 4, 7, 10, 18, 29, 34, and 38 cm on a side were used for this purpose. After this commissioning of the model, a 2D headscatter factor map (HSF) that varies as a function of spatial location can be generated for a given field's jaw settings. To complete the headscatter modeling a 2D relative fluence of a flood field $\left(\Psi_{\text{floa}}\right)$ was also generated (which is analogous to $F_{\text{horn}}$ in Jiang et al.^(24)^) To do this, dose was first calculated in the Eclipse TPS for a $40\times 40\;{\text{cm}}^{2}$ flood field in a water‐equivalent phantom, and subsequently deconvolved into a 2D flood field fluence using a 6 MV Monte Carlo pencil beam kernel scored in the same water phantom and geometry. This flood field fluence was then normalized on the central axis to create the relative 2D fluence flood field $\Psi_{\text{flood}}$ that includes the off‐axis horn structure. Since $\Psi_{\text{flood}}$ inherently contains inverse square effects from the TPS, no inverse square correction was applied to $\Psi_{\text{flood}}$ in the calculation of the HSF (as was done for $F_{\text{horn}}$ in Eq. (22) in Jiang et al.^(24)^). The final 2d HSF map (including the beam horns) was sampled at the same sampling grid as $\Psi_{\text{flood}}$ (500μm at isocenter). Multiplication of the $\Psi_{\text{flood}}$ and HSF maps created a 2D model of the fluence.

To obtain the EPID response from this 2D fluence, it was first geometrically magnified by a factor of 1.05 to match measurements made with the EPID at a source‐to‐image distance (SID) of 105 cm. The fluence was then resampled to match the EPID's pixel size and convolved in frequency space with an EPID response kernel $K_{\text{EPID}}$ (sampled at 784μm) to give a predicted 2D EPID image at 105 cm SID. $K_{\text{EPID}}$ is a radially symmetric kernel that accounts for the total (radiation and optical) scatter in the detector. It is generated from a sum of nine Gaussians, the parameters of which were optimized during commissioning of the Portal Dosimetry application of the Eclipse TPS.

The components of the basic 2D EPID response $S_{\text{basic}}$, in the absence of MLC interleaf leakage, transmission, and tongue‐and‐groove effects, are summarized in Eq. (3):

(3) $$D_{basic}=[\Phi_{ideas}\cdot HSF]⊗K_{EPID}$$

### C. Interleaf leakage and MLC transmission

**Figure 1:**
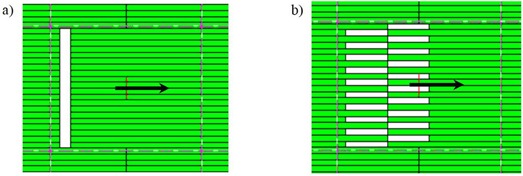
Illustrations depicting the beam's eye view (BEV) of a 1 cm sweeping‐window (a) and a 4 cm sweeping‐checkerboard (b) dynamic MLC delivery. The arrow shows the direction of leaf motion, where all fields are delivered over a $10\times 10\;{\text{cm}}^{2}$ field size defined by the collimator.

**Figure 2:**
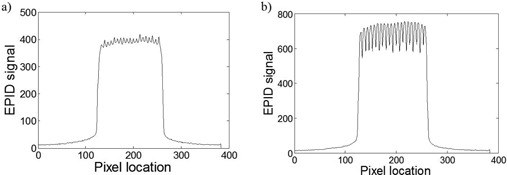
Profiles from EPID images taken with a $10\times 10\;{\text{cm}}^{2}$ field size and a 400 MU delivery. Profiles show the effects of a 1 cm sweeping‐window (a) and a 4 cm sweeping‐checkerboard (b) dynamic MLC delivery.

EPID acquisitions were made to characterize the 2D distribution of interleaf leakage alone. In order to quantify the shape of interleaf leakage across all MLCs, a 1 cm wide sweeping‐window MLC field was delivered to the EPID. This beam delivered uniform radiation over $10\times 30\;{\text{cm}}^{2}$ using 400 MUs. A dynamic MLC field was chosen, since it has been demonstrated that a sweeping‐window delivery (Fig. 1(a)) better characterizes interleaf leakage.^(21)^ Since none of the MLC leaves are staggered with respect to each other, the tongue‐and‐grove effect is not present. Instead, the EPID's response contains interleaf leakage (seen as the ‘peaks' in Fig. 2(a)) on some base signal. The EPID's response to this interleaf leakage alone was determined by removing the base from the peaks, and then scaling the interleaf leakage peaks such that their signal ranged from 0 to 1. This signal was then used as a template to determine a model of the averaged interleaf leakage profile in the Y direction (perpendicular to leaf travel) between all combinations of two neighboring leaves in 1D. The 1D profile is described by amplitudes at seven points, symmetric about a middle point of unit amplitude that is located at the center of the interleaf region between leaves. The amplitudes of the three points on either side are used to describe the falloff of the interleaf signal, and are sampled at the spacing of the EPID pixels. The 1D model was then applied over the X direction of MLC travel to create a 2D model $\left({\text{MLC}}_{\text{leakmaap}}\right)$ of the relative interleaf leakage. This model was extended to cover a $40\times 30\;{\text{cm}}^{2}$ field at the EPID pixel resolution, and took into account the two different leaf widths (0.5 cm and 1.0 cm) of the Millennium MLC. Because ${\text{MLC}}_{\text{leakmap}}$ defines the relative shape of just the interleaf leakage (normalized from 0 to 1) across the field, it is unaffected by any off‐axis effects which may be present in the EPID image due to beam softening or EPID arm scatter.

After the characterization of interleaf and transmission effects was complete, the basic EPID response $\left(S_{\text{basic}}\right)$ model described in the Material and Methods section B (above) could be extended to include these effects. Similar to the calculation of $\Phi_{\text{ideal}}$ (above), based on a given IMRT field's MLC leaf sequence, the interleaf template, ${\text{MLC}}_{\text{leakmap}}$ was applied to areas under the MLC leaves dynamically throughout the delivery to generate a field‐specific 2D map $\left({\text{MLC}}_{\text{leakmap}}{|}_{\;D}\right)$ of interleaf leakage. The amplitude of ${\text{MLC}}_{\text{leakmap}}{|}_{\;D}$ at any point in a field is equal to the relative fluence incident on (or ‘blocked’ by) an MLC leaf multiplied by the value of ${\text{MLC}}_{\text{leckmap}}$ at that point. For a 1 MU delivery it would range from 0 to 1: 0 for points never under the MLC leaves and/or lying in the middle of a leaf, and 1 for areas always under the leaves and midway between two leaves. A field‐specific 2D MLC transmission map $\left(T_{\text{MLC}}{|}_{\;D}\right)$ was also generated for each field. Since MLC transmission plus the ideal fluence equals unity, it was given a value of $1-\Phi_{\text{ideal}}$. So that the two dynamic maps could be added to $S_{\text{haSic}},\;{\text{MLC}}_{\text{leakmap}}{|}_{\;D}$ was multiplied by a weighting factor (A), and the EPID response kernel was deconvolved from it. This result was then added to the product of $T_{\text{MLC}}{|}_{\;D}$ and a weighting factor (B). The resulting sum was then multiplied by HSF and convolved with the EPID response kernel. Note that since ${\text{MLC}}_{\text{lekmap}}$ is already an EPID response, the EPID kernel must first be deconvolved from it prior to multiplication by the HSF. An EPID response that also incorporates interleaf leakage and transmission, $S_{L+T}$, is thus described by Eq. (4):

(4) $$S_{L+T}=S_{basic}+\left\{HSF\cdot \left[\left(A\cdot MLC_{leakmap}|{\;}_{D}\right){⊗}^{-1}\;K_{EPID}+B.T_{MLC}|{\;}_{D}\right]\right\}⊗K_{EPID}$$

The individual weighting factors corresponding to interleaf leakage (A) and transmission (B) in Eq. (4) were determined using a 1 cm sweeping‐window and an MLC‐covered $10\times 10\;{\text{cm}}^{2}$ field. An EPID image was acquired for a 400 MU sweeping‐window field, and the relative response $\left(S_{L+T}\right)$ was simulated with our MATLAB code. The weighting factor A was then optimized such that the relative magnitude of interleaf leakage was the same between the predicted $\left(S_{L+T}\right)$ and measured EPID responses. The weighting factor B was then optimized, such that the sum of both effects (interleaf leakage and transmission) were equal to that of our TPS's commissioning data (1.4%) when an MLC‐covered $10\times 10\;{\text{cm}}^{2}$ field was simulated.

### D. Tongue‐and‐groove effects

Similar to interleaf leakage, tongue‐and‐groove effect causes a 2D nonuniform change in dose within the delivered field. This effect must also be considered to provide an accurate dose prediction. Unlike interleaf leakage, tongue‐and‐groove effects decrease the dose only in the regions where adjacent MLC leaves are staggered. The magnitude of dose change at a point in the field depends on the fraction of delivery made by staggered leaf ends at that point. A worst‐case example can be seen with a 4 cm sweeping‐checkerboard field (Fig. 1(b)), where all of the MLC leaves are staggered (by 4 cm) during the entire delivery. The decrease in dose from tongue‐and‐groove effects can be very large (23% in Fig. 2(b)) compared to the increase in dose from interleaf leakage (5.7% in Fig. 2(a)).

To incorporate the tongue‐and‐groove effects of a field into our MATLAB simulations, a tongue‐and‐groove 2D response model was generated. The shape of the tongue‐and‐groove effect was characterized by four points (spaced at the EPID pixel resolution in the direction perpendicular to leaf travel) that were used to describe the dose reduction relative to an open field near the edge of an MLC leaf. Similar to ${\text{MLC}}_{\text{lekmap}}$, a 2D tongue‐and‐groove map ${\text{MLC}}_{T+G}$ covering a $30\times 40\;{\text{cm}}^{2}$ field was constructed by repeating this tongue‐and‐groove “penumbra” for all MLC leaves, and assuming the shape remained constant along the direction of leaf travel. This 2D map was then applied dynamically to a given IMRT field. At each of the 600 time points at which the IMRT field shape was sampled, the staggered leaf edges of the MLC were detected and were summed to produce a 2D cumulative image of the staggered leaf ends over the delivery. Multiplying this image by the ${\text{MLC}}_{T+G}$ template produced the field‐specific 2D map $({\text{MLC}}_{T+G}{|}_{\;D}$ of the tongue‐and‐groove effect. This tongue‐and‐groove component was applied to the $S_{\text{basic}}$ term in Eq. (4) through multiplication. The equation was then deconvolved using the EPID response kernel to yield the full fluence $\Phi_{\text{Full}}$.

(5) $$\Phi_{Full}=\left(S_{basic}\cdot MLC_{T+G}|{\;}_{D}\right){⊗}^{-1}K_{EPID}+HSF\cdot \left[\left(A\cdot MLC_{leakmap}|{\;}_{D}\right){⊗}^{-1}K_{EPID}+B\cdot T_{MLC}|{\;}_{D}\right]$$

The deconvolution in Eq. (5) was performed in frequency space using MATLAB. Negative or unphysical results were removed by rejecting any fluences outside of the field's jaw dimensions.

The amplitude of the four points describing the relative profile of the tongue‐and‐groove penumbra were optimized to give the best fit between the measured and simulated EPID images (obtained by convolving Eq. (5) with $K_{\text{EPID}}$) of the 4 cm sweeping‐checker (400 MUs, $10\times 10\;{\text{cm}}^{2}$) field. To validate the optimized shape of the tongue‐and‐groove penumbra, as well as the coefficients A and B, agreement between measured and simulated images of several (1, 2, and 4 cm) $10\times 10\;{\text{cm}}^{2}$ sweeping‐window and sweeping‐checker fields was confirmed. Once fully optimized and implemented into our code, $\Phi_{\text{Full}}$ provided a comprehensive means of predicting the fluence from any modulated field, including headscatter, interleaf leakage, transmission, and tongue‐and‐groove effects.

### E. Calculation of a 2D Modulation Map

Generation of dose to water used to calculate modulation factors required further processing of the full fluence. Monte Carlo pencil beam dose kernels $K_{\text{dose}}{|}_{\;D}$ were generated at a depth of interest, d, and convolved with the full fluence $\left(\Phi_{\text{Full}}\right)$ to generate dose in water at d. The doses for the modulated field and its corresponding open field were computed separately, and their ratio then taken to generate a 2D modulation map, $\text{Mod}{|}_{\;d}$:

(6) $$Mod|{\;}_{d}=\left[\left(\Phi_{Full}|{\;}_{\text{mod}}\right)⊗K_{dose}|{\;}_{d}\right]/\left[\left(\Phi_{Full}|{\;}_{open}\right)⊗K_{dose}|{\;}_{d}\right]$$

This 2D modulation map was used to calculate the modulation factor at the reference point in the beam's eye view (BEV) for each modulated field of an IMRT plan, at the depth d in water phantom geometry. For fields with a collimator rotation, the point was rotated with respect to the field, instead of rotating the 2D modulation map with respect to the measurement point.

### F. Experimental validation

To validate use of our 2D modulation map $\left(\text{Mod}{|}_{\;d}\right)$, modulation factors were measured using an ion chamber placed at a depth, d, in solid water at the same BEV position in the field as the plan's reference point. The measurement depth, d, for a specific field was the depth of the reference point in the patient. Since the modulation factor is not very depth‐dependant, for simplicity of measurement this depth was rounded to the nearest 5 cm. Modulation factors were also calculated using the 2D modulation map $\left(\text{Mod}{|}_{\;d}\right)$ for each field at the same depth. The patient's plan report and MLC files were exported to obtain the relevant beam parameters and reference point information. The map $\text{Mod}{|}_{\;d}$ was averaged over a 3.5mm×10.5mmROI centered at the reference point to emulate averaging of dose over the ion chamber's cylindrical active volume. To accommodate placement uncertainties of the ion chamber, the calculated value yielding the smallest percent discrepancy within a ±1mm ROI setup uncertainty (with respect to the reference point) was reported. A comparison of the simulated and measured modulation factors was made for 19 IMRT patient plans (121 fields) from a variety of sites: 14 prostate, 2 head‐and‐neck, 1 anal‐canal, 1 abdomen, and 1 hip. The IMRT fields included single‐, double‐, and triple‐split fields.

### G. Clinical Implementation

To calculate the 2D modulation maps $\left(\text{Mod}{|}_{\;d}\right)$ clinically at our center, an easy‐to‐use front end to our MATLAB code was developed, creating a new program Mod_Calc. This program included a GUI interface which prompts the user for inputs, and automatically calculates and compares modulation factor discrepancies between the Mod_Calc and Eclipse‐derived modulation factors. To reduce the time to calculate $\text{Mod}{|}_{\;d}$, all clinical simulations were performed with a spatial sampling of 1486μm at an isocenter, over 200 time intervals; this sampling was chosen to give approximately half the EPID sampling (750μm) at isocenter. Since our Eclipse plans generally use a $2.5\times 2.5\times 2.5\;{\text{mm}}^{3}$ calculation matrix, a $2.5\times 2.5\;{\text{mm}}^{2}$ ROI was averaged in Mod_Calc to determine a given modulation factor. In addition, since the location of any calculation point is only known to within half a pixel (1.25 mm) in Eclipse, the calculated value yielding the smallest percent discrepancy within a ±1.25mm ROI placement uncertainty (with respect to the reference point) is reported. This half‐pixel movement in the ROI calculation was made possible after resampling $\text{Mod}{|}_{\;d}$ (through linear interpolation) to 187μm.

The dosimetrist's clinical workflow of Mod_Calc is as follows. Once a treatment plan is completed, the dosimetrist exports the patient's plan report from Eclipse. For IMRT fields (including field‐in‐field) the beam's MLC file is also exported; the RapidArc plans require a DICOM plan export. For each modulated field, the dosimetrist also calculates the preliminary Eclipse‐based modulation factor (i.e., the dose for the modulated field at the reference point divided by the open‐field dose at the same point on the patient's dataset). The Mod_Calc program is then run, where it prompts the dosimetrist for the appropriate files. For each field in the plan, the BEV reference point location for the field's gantry and collimator angles is determined, as well as the radiological depth in the patient, rounded to the nearest centimeter. The modulation factor is calculated at this BEV location and depth for the modulated beam assuming normal incidence on a water phantom. Percentage differences between the modulation factors calculated by the Mod_Calc program and the Eclipse‐based prediction of the factors $(i.e.,\;\%\text{diff}=({\text{Mod}}_{\text{ModCalc}}-{\text{Mod}}_{\text{Eclipse}})/{\text{Mod}}_{\text{Eclipse}}\cdot 100\%$ are calculated and reported by Mod_Calc.

**Figure 3:**
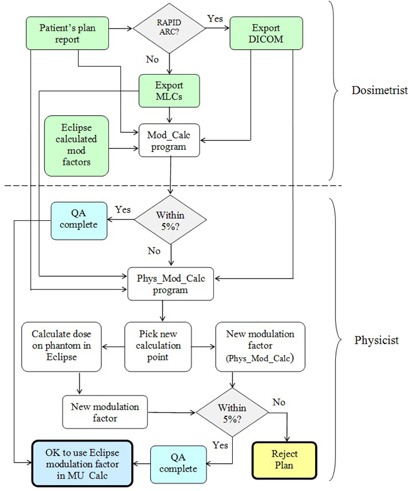
A flowchart showing the clinical workflow of the Mod_Calc program. Modulation factors are calculated based on the patients plan report, Eclipse‐calculated modulation factors, and MLC files (IMRT)/DICOM plan (RapidArc). The dotted line divides the tasks assigned to dosimetrists and physicists.

The physicist, during chart QA, checks that the reported modulation factor discrepancies are ≤ 5%, per our criterion. For fields that pass our criterion no action is required. However, when a discrepancy of >5% does occur, a second program, Phys_Mod_Calc, may be run to select a new reference point at which to perform the modulation factor comparison. This second program is identical to the Mod_Calc program, but it also displays a 2D image of $\left(\text{Mod}{|}_{\;10\text{cm}}\right)$ for the field being analyzed. Based on the location of the mouse arrow, at each click on the image, the modulation calculations are performed, and a corresponding location (with respect to isocenter) is reported at a default depth of 10 cm. This tool can be used to ensure that the new location of the reference point is in a high‐dose, low‐gradient region of the field. The modulation factor must then also be manually recalculated by the physicist in Eclipse at this new reference point. Unlike the original Eclipse‐based modulation calculation of ${\text{Mod}}_{\text{Eclipse}}$, which is calculated in patient geometry, the value of ${\text{Mod}}_{\text{Eclipse}}$ at the new reference point is recalculated in a “verification” plan at a depth of 10 cm (SSD 90 cm) on a water phantom to mimic the geometry of the Phys_Mod_Calc calculation. This eliminates any discrepancy present in the original comparison between Eclipse‐based and Mod_Calc factors that arose from differences in the geometry of the calculations. The new Phys_Mod_Calc modulation factors are manually compared to those derived from Eclipse to ensure the agreement satisfies our 5% criterion. If satisfied, the Eclipse‐predicted modulation factor is considered valid and can be used in the MUcalc calculation. In the case that this new well‐placed reference point fails our 5% criterion, the plan is rejected. A flow chart showing the clinical workflow of the two programs for dosimetrists and physicists is presented in Fig. 3.

Since its clinical implementation, results from the clinical version of Mod_Calc have been recorded in a file every time the program is run. The records include patient identifiers, the plan name, the Eclipse‐based and the Mod_Calc predictions of the modulation factor, and the percentage difference between the two values. This file has been useful for trending of the results, and for identifying any potential issues or limitations of the Mod_Calc program. For our analysis, this file was filtered to remove duplicate entries, as well as entries that were clearly erroneous. The latter occurred due to the rare transcription error of the Eclipse‐predicted modulation value, and when data were recorded for fields that were not intended to be evaluated. The latter case occurred, for instance, when only one field of a plan was to be recalculated at a new reference point, and “dummy” input was used for the other fields having passed the initial evaluation. After this filtering, the Mod_Calc “database” included 5271 entries within 923 plans (some patients had more than one plan).

## III. RESULTS

### A. Commissioning of Headscatter, Interleaf Leakage, Transmission, and Tongue and Groove

Optimization of the three Gaussian functions used in defining the relative intensity of the extrafocal source yielded the following parameters: $A_{1}=0.0805,\;A_{2}=0.0553,\;A_{3}=0.0523,\;\sigma_{1}=0.7552,\;\sigma_{2}=1.9982,\;\sigma_{3}=7.4928$ in Eq. (7). Here, x and y are the distances from isocenter (in cm) on the extrafocal plane.

(7) $$f(x,y)=\sum_{i=1}^{3}\frac{A_{i}}{2\pi \sigma_{i}^{2}}e^{-(x^{2}+y^{2})/2\sigma_{i}^{2}}$$

**Figure 4:**
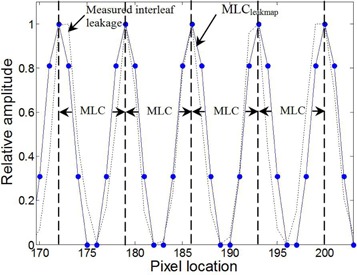
The relative amplitude of measured and modeled 2D interleaf leakage is shown across four MLC leaves. The edge of each MLC leaf is shown as a dashed line.

The 2D interleaf leakage $\left({\text{MLC}}_{\text{leakmap}}\right)$ response model can be seen in Fig. 4 for four MLC leaves, together with the measured interleaf leakage. It is normalized to unity and has a maximum value between MLC leaves. The ${\text{MLC}}_{\text{leakmiap}}$ is generated using amplitudes of 0, 0.31, 0.81, 1, 0.81, 0.31, and 0 to model the 1D shape of the interleaf effect between two leaves.

**Figure 5:**
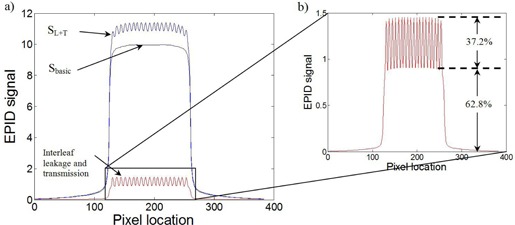
Simulation results showing the breakdown of the basic EPID model, interleaf leakage, and transmission for a 1 cm sweeping‐window $10\times 10\;{\text{cm}}^{2}$ field (a). The optimized weighting of interleaf leakage and transmission is also shown (b).

Simulation results following the optimization of weighting factors (A and B in Eq. (4)) obtained from the 1 cm sweeping‐window commissioning field are visualized in Figs. 5(a) and 5(b). The EPID response $S_{L+T}$ and its components $(S_{\text{basic}}$, interleaf leakage, and transmission) for the 1 cm sweeping‐window simulation are shown in Fig. 5(a). The values of A and B that provided the best fit to our experimental measurements were $6.054\times \;10^{-3}\;\text{and}\;1.014\times \;10^{-2}$, respectively, and the resulting interleaf and transmission magnitudes for a 1 cm sweeping‐window delivery are illustrated in Fig. 5(b). It should be noted that in this work, a single EPID response kernel was used in the optimization of factors A and B. Based on Greer et al.,^(25)^ beam‐hardening effects would produce a 2.2% error for our 1 cm sweeping‐window commissioning field. A transmission‐specific EPID kernel could be used to minimize this error. However, for typical clinical fields, the error would be reduced, as they are less modulated compared to this commissioning field.

**Figure 6:**
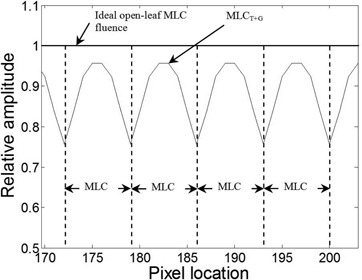
The relative amplitude of the ideal open‐MLC fluence and tongue‐and‐groove response is shown across four MLC leaves. The edge of each MLC leaf is shown as a dashed line.

The 2D tongue‐and‐groove $\left({\text{MLC}}_{T+G}\right)$ response model is illustrated in Fig. 6, along with the idealized open‐leaf MLC fluence $\left(\Phi_{\text{ideal}}\right)$ for four leaves. In contrast to interleaf leakage (Fig. 4), the tongue‐and‐groove profile has a minimum value between MLC leaves. The map is generated using penumbral amplitudes of 0.76, 0.84, 0.92, and 0.96, where the 0.76 is the amplitude at the edge of an extended, staggered MLC leaf, and the 0.96 corresponds to the amplitude at the midwidth location (for a 5 mm leaf) of the adjacent open MLC leaf.

### B. Experimental validation of $\Phi_{\text{Full}}$

**Figure 7:**
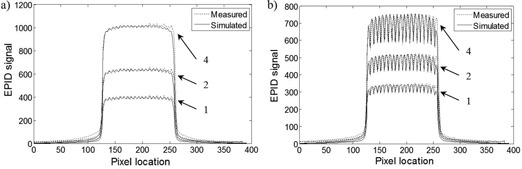
Simulated and measured EPID response profiles from 1, 2, and 4 cm sweeping‐window (a) and sweeping‐checker (b) $10\times 10\;{\text{cm}}^{2}$ fields.

Results from the full fluence model were verified by convolving $\Phi_{\text{Full}}$ with the EPID response kernel $K_{\text{EPID}}$ for a variety of fields, and comparing this predicted EPID response to the measured images. Comparisons for 1, 2, and 4 cm sweeping‐window and sweeping‐checker $10\times 10\;{\text{cm}}^{2}$ fields are seen in Figs. 7(a) and 7(b), respectively. These profiles perpendicular to the direction of leaf travel (along the central axis) show excellent agreement between the simulated and the measured fields. Important to note is that the shape and magnitude of interleaf leakage and tongue‐and‐groove effects are properly scaled, regardless of the gap size or length of stagger of the dynamic delivery. The simulated EPID responses seen in Fig. 7 were all multiplied by a common scaling factor to account for differences between the raw EPID signal values and the simulated signal values.

### C. Modulation factors: simulated vs. measured

**Figure 8:**
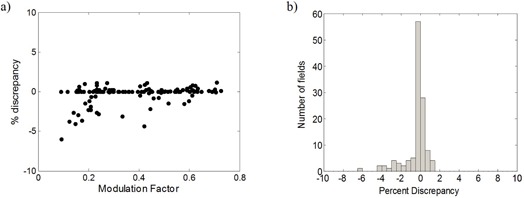
Percent discrepancy between Mod_Calc‐simulated and ion chamber‐measured modulation factors over a range of modulation factors (a), and a histogram of the percent difference between the two (b).

The percent discrepancy between measured modulation factors and Mod_Calc can be seen in Figs. 8(a) and 8(b). The scatter plot in Fig. 8(a) shows good agreement between the two, regardless of the magnitude of the modulation factor, with larger discrepancies found for smaller modulation factors. A histogram of the percent difference between the Mod_Calc and measurement (normalized to the measured value) is presented in Fig. 8(b). Again there is very good agreement: over the 121 fields measured, the mean difference is −0.3%, with a standard deviation of 1.2%. Retrospective analysis of the data revealed that the larger discrepancies appearing in Fig. 8 were due to the placement of the reference/measurement point in a high‐gradient region. The Mod_Calc calculation time for each field was 55 sec on a single core processor.

### D. Modulation factors: clinical results and experience

**Figure 9:**
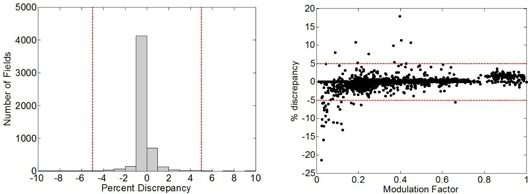
Percent discrepancy between Mod_Calc and Eclipse‐based predictions of the modulation factor (Mod_Calc ‐ Eclipse) for all entries in the database file (a) frequency distribution of the discrepancy; (b) scatter plot of the percent discrepancy values with respect to the Eclipse‐based modulation factor value.

Figure 9(a) shows a histogram of the percentage discrepancy between the Mod_Calc and Eclipse‐based predictions of the modulation factor, illustrating the agreement between the two values. The percentage of entries falling outside our clinical 5% agreement criterion was 0.7% (37 of 5271); 3.5% of the entries were ≤ 2% discrepancy. Figure 9(b) presents a scatter plot of the discrepancy as a function of the Eclipse‐predicted Mod value. This illustrates that the majority of the 37 entries outside 5% tolerance belonged to fields with a low modulation factor. Most often these cases corresponded to a reference point falling in a low‐dose region, where scatter and/or MLC transmission contributions are larger than usual. This is not uncommon, since it is not always possible to select a reference point location that is optimal for all fields of an IMRT plan. Since a limitation of our TPS is its treatment of MLC interleaf leakage and tongue and groove, the TPS prediction is expected to be more uncertain for low dose points. The larger discrepancies between the TPS and Mod_Calc predictions for such points were, thus, not particularly surprising. Neither were the discrepancies generally troubling, since large discrepancies at low modulation points usually correspond to a small absolute dose difference, provided that other parts of the field and/or plan have significantly higher modulation factors. The number of low modulation points flagged by Mod_Calc would have been reduced had an alternative normalization of the % discrepancy been used — for example, calculating the discrepancy as a percentage of the maximum modulation in the field or plan, rather than with respect to the modulation at the given point, as we have done.

Typically in scenarios where large discrepancies are flagged, the physicist would follow one of two courses of action. For the specific field(s) with poor agreement, a Mod_Calc/TPS comparison can be calculated for a reference point located in a higher dose/low gradient region. The second option, which became more frequent as experience with such points was gained, was to accept the discrepancy without further action if the 2D portal dosimetry analysis did not indicate any concerns.

**Figure 10:**
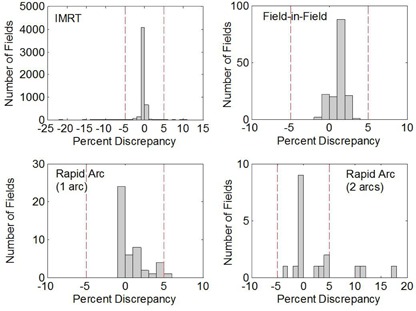
Frequency distribution of the percent discrepancy (Mod_Calc ‐ Eclipse) in the modulation factor predictions as a function of the type of dynamic delivery.

Figure 10 and Table 1 provide further analysis of subsets of the Mod_Calc database based on the type of dynamic delivery and treatment site. Since the majority (5053 of 5271) of the entries is for the sliding‐window IMRT fields, the frequency histogram and corresponding statistics are nearly the same as for the entire database (cf. Fig. 9). A large percentage (3686 of 5053) of the IMRT entries are reported as having no discrepancy (recall that the Mod_Calc reports the minimum discrepancy within a ±1.25mm ROI of the reference point location). The mean of the absolute value of the discrepancy, $\overset{―}{\left|\%\text{diff}\right|}$, is 0.3%. Of the 33 fields that exceeded the 5% tolerance, nine had Eclipse modulation factors greater than 0.12 at the original reference point. Due to the large number of IMRT entries, these could be further categorized by treatment site based on examination of the plan name for each entry: GI‐GU (3396 entries), head‐and‐neck (679), brain/CNS (286), and breast (99). The 593 “uncategorized” entries were those for which the treatment site was different than these four sites, or could not be readily identified from the plan name. In general, there is little statistical difference between the sites, with the exception of the breast entries. For each of the GI‐GU (mainly prostate), head‐and‐neck, brain/CNS, and uncategorized subsets, the $\overset{―}{\left|\%\text{diff}\right|}$ was less than 0.5%. The percentage of entries where $\overset{―}{\left|\%\text{diff}\right|}$ was more than 5% and 2% was less than 1% and 7%, respectively. The statistics for the relatively small number of breast entries differed, with the $\overset{―}{\left|\%\text{diff}\right|}$ being 1.7%, and eight of the 99 fields had a $\overset{―}{\left|\%\text{diff}\right|}$ greater than 5%. Nevertheless, of the eight “failures,” six were for fields with modulation factors less than 0.12, and the other two were from a single plan. Overall, the agreement between Mod_Calc and the Eclipse‐based predictions of the modulation factor has still been acceptable for the breast IMRT fields, and extensive investigation of the source of the slightly worse agreement (compared to other sites) has not been warranted to this point.

**Table 1:** Statistics for the database entries as a function of dynamic delivery type and treatment site (for IMRT deliveries). For each category, $\overset{―}{\text{Mod}}$ and $\sigma_{\text{Mod}}$ represent the mean and standard deviation of the modulation factors, respectively; $\overset{―}{\left|\%\text{diff}\right|}$ and $\sigma_{\%\text{diff}}$ are the mean of the absolute value and the standard deviation of the percentage discrepancy between the Mod_Calc and Eclipse‐based values; and the final two columns quantify the percentage of the fields in each category where the percentage discrepancy is less than 5% and 2%, respectively

*Type of Field*	*No. of Fields*	*No. of Plans*	$\overset{―}{\text{Mod}}$	$\sigma_{\text{Mod}}$	$\left|\overset{―}{\%\text{diff}}\right|$	$\sigma_{\%\text{diff}}$	*Percent of Fields with* |%diff|<5%	*Percent of Fields with* |%diff|<2%
All	5271	923	0.38	0.19	0.3	1.0	99.3	96.5
IMRT	5053	779	0.36	0.17	0.2	0.9	99.3	97.2
GI‐GU	3396	3396	0.38	0.17	0.2	0.7	99.5	98.3
Head & Neck	679	114	0.29	0.15	0.4	1.4	99.0	93.7
Brain	286	54	0.44	0.13	0.1	0.2	100.0	100.0
Breast	99	24	0.28	0.16	1.7	3.2	91.9	72.7
Uncategorized	593	94	0.34	0.16	0.2	0.6	99.7	97.5
Field‐in‐Field	154	89	0.90	0.08	1.3	0.9	100	85.7
Rapid Arc (1 arc)	46	46	0.48	0.05	1.0	1.5	97.8	82.6
Rapid Arc (2 arcs)	18	9	0.44	0.10	3.3	5.4	83.3	55.6

Although Mod_Calc was initially written to compute the modulation factor for IMRT fields, as clinical need has arisen, it has been modified to also accommodate other modulated deliveries. These include step‐and‐shoot field‐in‐field, and by adding gantry‐dependent dose rates to the program, RapidArc deliveries are also supported. At our center, field‐in‐field beams are used for some of our two‐field conformal breast plans to improve dose homogeneity. For the 154 entries analyzed, $\left|\overset{―}{\%\text{diff}}\right|$ was 1.3%, and the frequency histogram (Fig. 10) shows a systematic shift to the right, indicating that Mod_Calc predicts slightly larger modulation values. Regardless, thus far, none of the field‐in‐field fields have exceeded our clinical tolerance of 5%, and all but one have been within 3%.

Our experience with RapidArc is preliminary. Clinical implementation at our center occurred in July of 2012 for one‐arc treatments, and in September for two‐arc deliveries. Thus far, RapidArc has only been used for prostate treatments. A majority of the fields show good agreement, with 31 of the 55 RapidArc entries (one and two arcs) having no discrepancy. However, the preliminary results also show a higher frequency of larger discrepancies, and 4 of the 55 fields exceed the 5% criterion. Based on the limited sample size, the two‐arc plans appear more problematic, with three of 18 fields failing. These failures could not be attributed to the field having a low modulation value. Further investigation has suggested that the Mod_Calc comparison is quite sensitive to the location of the reference point. Analysis has suggested that locating the reference point in a high‐gradient region caused these failures. This is more likely for two‐arc deliveries: the reference point is selected based on the combined dose of both arcs, but this location may be in a gradient region for an individual arc. Failures may also occur when the reference point is in a region where the deficiencies in the modeling of tongue‐and‐groove effects by our TPS are exacerbated. This may occur when the point is near an MLC leaf that is extended apart from its leaf neighbors for a significant part of the delivery. When failures for the RapidArc fields have occurred, a new reference point has been selected with the aid of the Phys_Mod_Calc program, and the subsequent agreement between the Mod_Calc and Eclipse‐based modulation factors has been acceptable.

## IV. DISCUSSION

Results from the experimental validation of Mod_Calc and comparison with similar validation of previous pencil beam algorithms have given us confidence in the clinical suitability of the Mod_Calc algorithm. Clinical evaluation by Georg et al.^(17)^ of their “MUV” verification software reports mean discrepancies between doses calculated in a homogeneous phantom and ion chamber (IC) measurements of −0.7%±4.1%(1SD)and0.6%±2.5% based on analysis of 56 dynamic and 48 step‐and‐shoot IMRT fields, respectively. A similar validation of the algorithm (dose at 15 cm depth in phantom) by Azcona and Burguete^(15)^ for 35 step‐and‐shoot fields yielded a discrepancy of 1.2%±3.5%. (These numbers are based on the results found in Table II^(15)^ and analysis with respect to the local point dose, as opposed to the maximum dose, as was done originally). For 121 dynamic IMRT fields, we obtained a mean difference between Mod_Calc and measurement of −0.3%±1.2%. Due to specifics of the analyses, our smaller reported standard deviation should not necessarily be interpreted as achievement of greater accuracy. For example, in general, the reported results will depend on the specific cohort of fields tested (e.g., their degree of modulation and the selected location of the calculation points). Our quoted results are also based on the minimum discrepancy within a ±1mm ROI, to account for uncertainties in IC positioning. Using an ROI window will, of course, reduce discrepancies, especially in high‐gradient regions. A similar ROI approach is used by Azcona and Burguete, but (apparently) not employed by Georg et al. (The latter do, however, ensure that the point‐dose comparisons are performed in low‐gradient regions). Despite these caveats, our validation results are at least comparable to these earlier reports, and suggest sufficient accuracy of our algorithm.

As done in this work, previous studies also compared their independent dose calculations to TPS calculations. Watanabe^(5)^ reported agreement within ±2% when calculating at the isocenter of prostate IMRT plans, provided isocenter was located in a low‐gradient region. This was achieved despite the limitations of the algorithm — use of a spatially‐invariant kernel, and lack of modeling of head scatter and MLC interleaf and tongue‐and‐groove effects. Larger discrepancies were observed for head‐and‐neck treatments and in high‐gradient regions. Azcona and Burguete^(15)^ compared their doses at 15 cm depth in a polystyrene phantom to those calculated by the pencil beam algorithm of their KonRad TPS in the same geometry. The mean dose difference (relative to the maximum dose in the plane, as opposed to the local dose) for 541 individual step‐and‐shoot IMRT fields was −0.4%±1.8% (1 SD), with a range of −6.9%to6.5%. The magnitudes of the dose differences were relatively independent of tumor site. Our analysis of 5053 dynamic IMRT fields yielded a similar mean value of 0.2%, but a much smaller standard deviation of 0.9%. We also did not find large variations in the results between tumor sites, with the exception of the breast IMRT fields, and to a much lesser extent, head‐and‐neck fields. The MUV algorithm, which incorporates a spatially variant kernel, was benchmarked with respect to the clinical pencil beam algorithms (Helax TMS and Oncentra MasterPlan; Nucletron BV, Veenendaal, The Netherlands) in Georg et al.^(12)^ A dose difference (in phantom geometry) of 1.1%±2.9% was obtained for 367 step‐and‐shoot IMRT plans. Although strict comparisons between the different studies is not possible (due to specifics of the analyses) or even particularly meaningful (due to differing reference TPS's), our results do further support the clinical utility of our Mod_Calc program. The source of the somewhat tighter agreement between Mod_Calc and our TPS and these other studies is not easy to identify, and was not investigated further.

In addition to accuracy, (modest) complexity and commissioning requirements were secondary criteria for the development of our method. For this assessment, it is helpful to consider the algorithm's fluence modeling and kernel components separately. To summarize, our fluence model uses: a characterization of off‐axis fluence (i.e., $\Psi_{\text{flood}}$); the head scatter model of Jiang et al.,^(24)^ which requires a fit of collimator scatter data to extract six parameters for the extrafocal source characterization; and the MLC file of each field to calculate the modulated primary fluence. The modeling of MLC‐specific effects on fluence requires an average MLC transmission factor, a dosimetric leaf gap parameter to account for rounded leaf ends, and three EPID images to extract the two parameters (A and B in Eq. (4)) and the 2D maps $\left({\text{MLC}}_{\text{leakmap}}\;\text{and}\;({\text{MLC}}_{T+G}\right)$ needed to model interleaf leakage and tongue‐and‐groove effects. Although many details of the implementations differ, on sum, the fluence modeling complexities of our algorithm and those of Azcona and Burguete^(18)^ and Oloffson et al.^(26)^ (MUV software) are comparable. These two earlier algorithms also incorporate an analytic modeling of the headscatter fluence that relies on commissioning data (output factors). With respect to MLC effects, Georg et al.^(12)^ state that the MUV software incorporates rounded leaf ends and tongue and groove, but do not provide details. Azcona and Burguete^(15)^ rely on the analytic MLC modeling of Chui et al.,^(27)^ which provides a more sophisticated spatial characterization than our approach with respect to the rounded leaf ends, but less so with respect to the tongue‐and‐groove perturbations. It is also noted that our algorithm's modeling is superior to that of our TPS, which ignores interleaf leakage and approximates tongue‐and‐groove effects as an equivalent uniform reduction in fluence over the entire width of an affected MLC leaf. For example, unlike our MLC model where the spatially varying tongue‐and‐groove map (Fig. 6) is applied, the dose under a pair of open MLC leaves modeled by the TPS is reduced uniformly by approximately 8%‐10% if the neighboring leaf pair on one side is closed. This can result in as much as a 10% overestimation of dose near the leaf edge, and a 10% underestimation of dose in the middle of the two open leaves.

The treatment of the pencil beam kernel is a significant distinction between our approach and earlier ones. Our kernel is generated at various depths using Monte Carlo simulations using a published linac spectrum.^(22)^ No commissioning of the kernel is performed. Generation of a spatially invariant kernel at a given depth in Azcona et al.^(^ ^15^ ^,^ ^18^ ^)^ is more complex, involving a Hankel transform calculation of 2D film data, and refinement based on measured output factor data. The depth‐dependent kernel used in the MUV software is described by Nyholm et al.^(^ ^19^ ^,^ ^28^ ^)^ It requires only one input commissioning datum $\left({\text{TPR}}_{20,10}\right)$, but uses a rather complicated mathematical expression involving 102 parameters. This kernel model is the most sophisticated, having been extended to include off‐axis changes in energy spectrum using the method developed by Nyholm et al.^(28)^ The spatial variance of this kernel is a potentially significant advantage of the MUV software algorithm. Olofsson et al.^(29)^ quote a potential increase in accuracy of pencil beam dose calculations of up to 4% (at 20 cm depth and 18 cm off‐axis) when the kernel accounts for off‐axis spectral softening. However, the results from validation and use of Mod_Calc suggest that the spatial invariance of our kernel is not a significant limitation. Although not specifically quantified, one possible reason for this is that few of the calculation points included in our analysis are more than 10 cm off‐axis. More significantly, in general, the calculation of modulation factors will tend to reduce off‐axis sensitivity, since inaccuracies in the IMRT dose at an off‐axis point will be largely canceled by similar inaccuracies in the calculation of the open‐field dose. In our second check dose calculation, off‐axis softening is included via the depth‐dependent OAR in Eq. (1) used by the MUcalc calculation.

An alternative to using our algorithm to calculate IMRT modulation factors would be its use for direct calculation of the point dose. Intuitively this would seem preferable: it would obviate use of a second computer program (in our case, MUcalc) and provide a more complete assessment of the total discrepancies with TPS calculations. However, it can be argued that the benefits may not be as profound as they first appear. Generally, independent IMRT dose calculations are done in water phantom geometry. In this case, the conceptual difference between calculating dose and modulation is relatively minor — the two quantities are related by an open‐field water phantom dose, which should be well described by both the TPS and any second‐check software. An independent IMRT dose calculation attempting to incorporate patient geometry could use a kernel that is evaluated at the radiological depth of each calculation point.^(17)^ Although more comprehensive, this more advanced approach would also come at the cost of making it more difficult to identify the source of concerning discrepancies between TPS and independent dose calculations (e.g., MLC‐related effects or inhomogeneities). This highlights a potential advantage for troubleshooting of separate modulation factor calculation. As implied by the above discussion regarding the spatial variance of the kernel, another potential benefit of this separation is that it may reduce the required model complexity and/or commissioning requirements of the algorithm of the IMRT‐dependent part of the verification calculation. In this way, the extensive commissioning of an existing second‐check calculation system for conformal treatments (e.g., MUcalc) can be exploited.

## IV. CONCLUSIONS

The development of Mod_Calc has provided a robust method of calculating modulation factors, which is an important component of the independent verification of MUs for dynamic fields at our clinic. The method implements a comprehensive 2D fluence model that incorporates MLC interleaf leakage, transmission, and tongue‐and‐groove effects to an extent that surpasses the modeling of these effects by our clinical TPS. The parameters used to characterize these MLC effects in our model are commissioned using EPID images of a small number of fields. A comparison between the predictions of Mod_Calc and measurements using ion chambers of the modulation factors of 121 IMRT fields yielded excellent agreement, with a mean difference (Mod_Calc ‐ ion chamber) of −0.3%±1.2%. This agreement gave us confidence to implement the method clinically. The modulation factors calculated using our TPS are now verified using Mod_Calc calculation, rather than the onerous ion chamber measurements that were used previously. An analysis of the database records of 5271 dynamic fields delivered at our clinic indicates that the mean difference between the Mod_Calc and TPS‐generated predictions is 0.3%±1%. Additionally, 99.3% of these fields passed our clinical criterion requiring agreement within 5%, and 96.5% of the fields agreed within 2%. Since we no longer need to perform ion chamber measurements for the verification of modulation factor values, our clinical quality assurance process for these dynamic fields has been streamlined considerably. Our improved in‐house method of MU verification has removed the impetus to resort to commercial software alternatives.

## Supporting information

Supplementary Material

Supplementary Material

## References

[1] Stern RL , Heaton R , Fraser MW , et al. Verification of monitor unit calculations for non‐IMRT clinical radiotherapy: report of AAPM Task Group 114. Med Phys. 2011;38(1):504–30. 21361219 10.1118/1.3521473

[2] Low DA , Moran JM , Dempsey JF , Dong L , Oldham M . Dosimetry tools and techniques for IMRT. Med Phys. 2011;38(3):1313–38. 21520843 10.1118/1.3514120

[3] Kung JH , Chen GTY , Kuchnir FK . A monitor unit verification calculation in intensity modulated radiotherapy as a dosimetry quality assurance. Med Phys. 2000;27(10):2226–30. 11099189 10.1118/1.1286553

[4] Xing L and Li JG . Computer verification of fluence map for intensity modulated radiation therapy. Med Phys. 2000;27(9):2084–92. 11011737 10.1118/1.1289374

[5] Watanabe Y . Point dose calculations using an analytical pencil beam kernel for IMRT plan checking. Phys Med Biol. 2001;46(4):1031–38. 11324949 10.1088/0031-9155/46/4/309

[6] Chen Z , Xing L , Nath R . Independent monitor unit calculation for intensity modulated radiotherapy using the MIMiC multileaf collimator. Med Phys. 2002;29(9):2041–51. 12349925 10.1118/1.1500397

[7] Yang Y , Xing L , Li JG , et al. Independent dosimetric calculation with inclusion of head scatter and MLC transmission for IMRT. Med Phys. 2003;30(11):2937–47. 14655941 10.1118/1.1617391

[8] Linthout N , Verellen D , Van Acker S , Storme G . A simple theoretical verification of monitor unit calculation for intensity modulated beams using dynamic mini‐multileaf collimation. Radiother Oncol. 2004;71(2):235–41. 15110458 10.1016/j.radonc.2004.02.014

[9] Popescu IA , Shaw CP , Zavgorodni SF , Beckham WA . Absolute dose calculations for Monte Carlo simulations of radiotherapy beams. Phys Med Biol. 2005;50(14):3375–92. 16177516 10.1088/0031-9155/50/14/013

[10] Baker CR , Clements R , Gately A , Budgell GJ . A separated primary and scatter model for independent dose calculation of intensity modulated radiotherapy. Radiother Oncol. 2006;80(3):385–90. 16956682 10.1016/j.radonc.2006.08.011

[11] Fan J , Li J , Chen L , et al. A practical Monte Carlo MU verification tool for IMRT quality assurance. Phys Med Biol. 2006;51(10):2503–15. 16675866 10.1088/0031-9155/51/10/010

[12] Georg D , Stock M , Kroupa B , et al. Patient‐specific IMRT verification using independent fluence‐based dose calculation software: experimental benchmarking and initial clinical experience. Phys Med Biol. 2007;52(16):4981–92. 17671348 10.1088/0031-9155/52/16/018

[13] Lin MH , Chao TC , Lee CC , Tung CJ , Yeh CY , Hong JH . Measurement‐based Monte Carlo dose calculation system for IMRT pretreatment and on‐line transit dose verifications. Med Phys. 2009;36(4):1167–75. 19472622 10.1118/1.3089790

[14] Pisaturo O , Moeckli R , Mirimanoff RO , Bochud FO . A Monte Carlo‐based procedure for independent monitor unit calculation in IMRT treatment plans. Phys Med Biol. 2009;54(13):4299–310. 19531844 10.1088/0031-9155/54/13/022

[15] Azcona JD and Burguete J . Intensity modulated dose calculation with an improved experimental pencil‐beam kernel. Med Phys. 2010;37(9):4634–42. 20964182 10.1118/1.3476467

[16] Yang Y , Xing L , Boyer AL , Song YX , Hu YM . A three‐source model for the calculation of head scatter factors. Med Phys. 2002;29(9):2024–33. 12349923 10.1118/1.1500767

[17] Georg D , Nyholm T , Olofsson J , et al. Clinical evaluation of monitor unit software and the application of action levels. Radiother Oncol. 2007;85(2):306–15. 17904234 10.1016/j.radonc.2007.04.035

[18] Azcona JD and Burguete J . A system for intensity modulated dose plan verification based on an experimental pencil beam kernel obtained by deconvolution. Med Phys. 2008;35(1):248–59. 18293580 10.1118/1.2815359

[19] Nyholm T , Olofsson J , Ahnesjo A , Karlsson M . Photon pencil kernel parameterisation based on beam quality index. Radiother Oncol. 2006;78(3):347–51. 16515812 10.1016/j.radonc.2006.02.002

[20] Steciw S , Warkentin B , Rathee S , Fallone B . A comprehensive MLC dose model: use in dynamic IMRT verification using an EPID [abstract]. Med Phys. 2009;36(6):2566.

[21] Rosca F and Zygmanski P . An EPID response calculation algorithm using spatial beam characteristics of primary, head scattered and MLC transmitted radiation. Med Phys. 2008;35(6):2224–34. 18649452 10.1118/1.2911870

[22] Sheikh‐Bagheri D and Rogers DW . Monte Carlo calculation of nine megavoltage photon beam spectra using the BEAM code. Med Phys. 2002;29(3):391–402. 11930914 10.1118/1.1445413

[23] Vial P , Oliver L , Greer PB , Baldock C . An experimental investigation into the radiation field offset of a dynamic multileaf collimator. Phys Med Biol. 2006;51(21):5517–38. 17047267 10.1088/0031-9155/51/21/009

[24] Jiang SB , Boyer AL , Ma CM . Modeling the extrafocal radiation and monitor chamber backscatter for photon beam dose calculation. Med Phys. 2001;28(1):55–66. 11213923 10.1118/1.1333747

[25] Greer PB , Vial P , Oliver L , Baldock C . Experimental investigation of the response of an amorphous silicon EPID to intensity modulated radiotherapy beams. Med Phys. 2007;34(11):4389–98. 18072504 10.1118/1.2789406

[26] Olofsson J , Georg D , Karlsson M . A widely tested model for head scatter influence on photon beam output. Radiother Oncol. 2003;67(2):225–38. 12812855 10.1016/s0167-8140(02)00409-7

[27] Chui C , LoSasso T , Palm A . A practical guide to intensity‐modulated radiation therapy. Madison, WI: Medical Physics; 2003.

[28] Nyholm T , Olofsson J , Ahnesjo A , Karlsson M . Modelling lateral beam quality variations in pencil kernel based photon dose calculations. Phys Med Biol. 200621;51(16):4111–18. 16885628 10.1088/0031-9155/51/16/016

[29] Olofsson J , Nyholm T , Georg D , Ahnesjo A , Karlsson M . Evaluation of uncertainty predictions and dose output for model‐based dose calculations for megavoltage photon beams. Med Phys. 2006;33(7):2548–56. 16898459 10.1118/1.2207316

