# Supplementary figures and images for: Modulation factors calculated with an EPID‐derived MLC fluence model to streamline IMRT/VMAT second checks
Source: J Appl Clin Med Phys. 2013 Nov 8;14(6):62–81. doi: 10.1120/jacmp.v14i6.4274 (PMC5714641; doi:10.1120/jacmp.v14i6.4274)

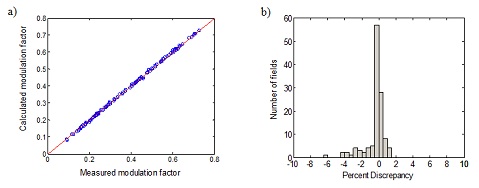

Supplement: Supplementary file 1 — Supplementary Material [file ACM2-14-062-s001.jpg]

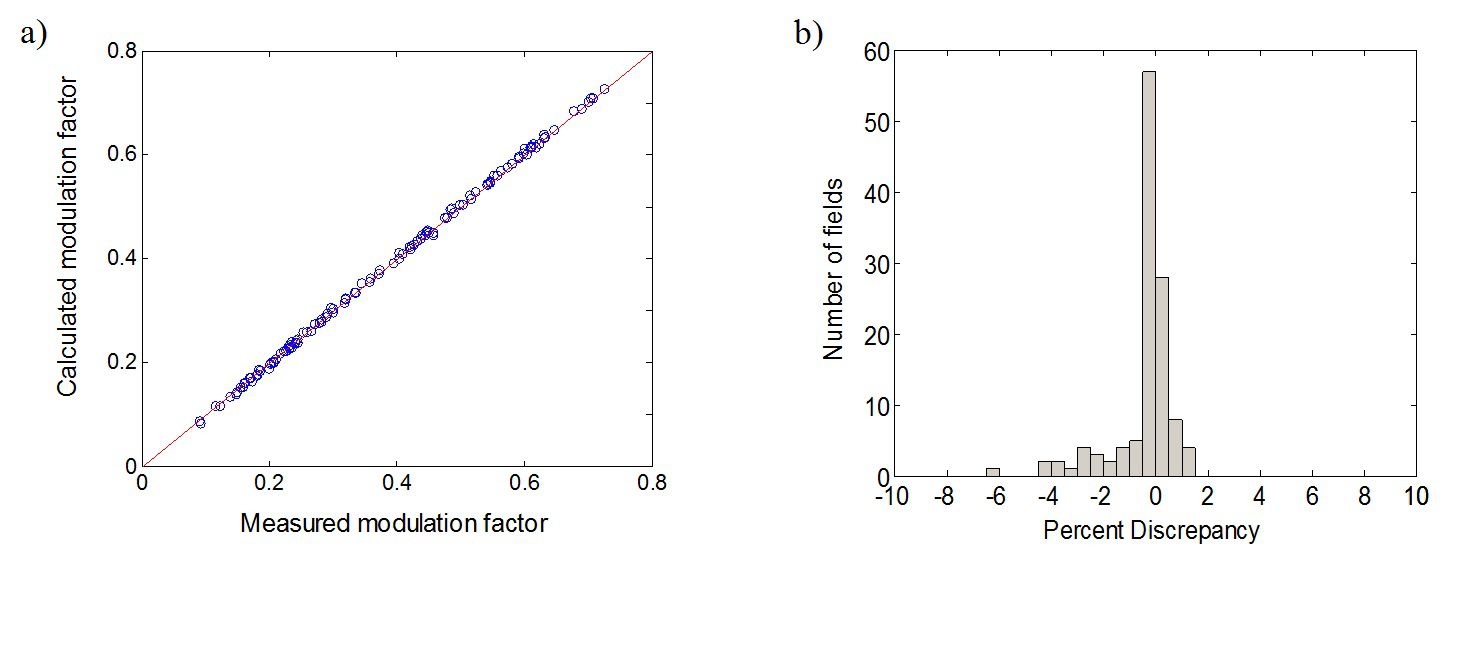

Supplement: Supplementary file 2 — Supplementary Material [file ACM2-14-062-s002.jpg]
